# Exploring the link between estimated glucose disposal rate and Parkinson’s disease: cross-sectional and mortality analysis of NHANES 2003–2016

**DOI:** 10.3389/fnagi.2025.1548020

**Published:** 2025-04-04

**Authors:** Xiaoting Li, Zhaohao Zeng

**Affiliations:** ^1^Department of Neurology, Panyu Hexian Memorial Affiliated Hospital of Guangzhou, Guangzhou, China; ^2^Department of Neurology, Shenzhen People’s Hospital (The First Affiliated Hospital, Southern University of Science and Technology, The Second Clinical Medical College, Jinan University), Shenzhen, Guangdong, China; ^3^Guangdong Provincial Clinical Research Center for Geriatrics, Shenzhen Clinical Research Center for Geriatrics, Shenzhen People’s Hospital (The Second Clinical Medical College, Jinan University, The First Affiliated Hospital, Southern University of Science and Technology), Shenzhen, China

**Keywords:** Parkinson’s disease, estimated glucose disposal rate, insulin resistance, NHANES, all-cause mortality

## Abstract

**Objectives:**

To investigate the association between estimated glucose disposal rate (eGDR), a surrogate marker of insulin resistance, and Parkinson’s disease (PD) risk, and to examine the relationship between eGDR and all-cause mortality among PD patients.

**Methods:**

Using data from the National Health and Nutrition Examination Survey (NHANES) 2003–2016, we conducted a cross-sectional study of 20,767 participants aged ≥40 years. eGDR was calculated using waist circumference, hypertension status, and HbA1c levels. PD cases were identified through anti-parkinsonian medication use. The association between eGDR and PD was examined using weighted logistic regression models with progressive adjustment for potential confounders. Survival analysis was performed in 255 PD patients to assess the relationship between eGDR and all-cause mortality.

**Results:**

Among participants, 256 had PD (weighted prevalence: 1.23%). Higher eGDR was associated with lower odds of PD in crude analysis (OR: 0.906, 95% CI: 0.856–0.960, *P* < 0.001). After full adjustment, the highest eGDR tertile showed significantly lower odds of PD compared to the lowest tertile (OR: 0.574, 95% CI: 0.337–0.976, *P* = 0.040). Restricted cubic spline analysis revealed a significant M-shaped non-linear relationship between eGDR and PD risk (P for non-linearity < 0.001). In survival analysis, higher eGDR was associated with lower mortality risk (adjusted HR: 0.875, 95% CI: 0.775–0.987, *P* = 0.030), with an inverted U-shaped relationship observed (P for non-linearity = 0.0352).

**Conclusion:**

Higher eGDR levels are associated with lower PD risk and better survival in PD patients, suggesting that insulin sensitivity might play a role in PD pathogenesis and progression. These findings highlight the potential importance of metabolic health in PD.

## 1 Introduction

Parkinson’s disease (PD) is one of the most common neurodegenerative disorders, characterized by motor and non-motor symptoms, affecting approximately 1% of individuals aged over 60 years worldwide (Shtilbans and Henchcliffe, 2012; Maciotta et al., 2013). Emerging research suggests that metabolic dysfunction may contribute to PD development and progression (Dai et al., 2023; Ye et al., 2023; Zhong et al., 2024). Among various metabolic factors, insulin resistance has emerged as a significant area of interest in PD research (Athauda and Foltynie, 2016).

Insulin resistance, traditionally associated with type 2 diabetes and cardiovascular disease, has been implicated in various neurodegenerative processes (Athauda and Foltynie, 2016; Hölscher, 2020). Studies have shown that insulin signaling plays crucial roles in neuronal survival, synaptic plasticity, and brain glucose metabolism (Kullmann et al., 2016; Kellar and Craft, 2020). Disruption of insulin signaling in the central nervous system has been linked to increased neurodegeneration and cognitive decline (Kullmann et al., 2016). Several epidemiological studies have reported associations between type 2 diabetes, insulin resistance, and increased risk of PD (Yue et al., 2016; Cheong et al., 2020; Aune et al., 2023), although the results have been inconsistent (Simon et al., 2007; Palacios et al., 2011).

The estimated glucose disposal rate (eGDR), originally developed to assess insulin sensitivity in type 1 diabetes (Williams et al., 2000), has emerged as a valuable surrogate marker for insulin resistance. This index, calculated from routinely measured clinical parameters (waist circumference, hypertension, and HbA1c), serves as a practical and scalable tool for assessing insulin sensitivity in large-scale epidemiological studies, circumventing the need for complex laboratory procedures like hyperinsulinemic-euglycemic clamps, and extending its relevance beyond diabetes research to fields such as neurodegenerative disease investigations (Guo et al., 2024; He et al., 2024; Peng et al., 2024). While eGDR has been extensively studied in diabetes and cardiovascular diseases (Williams et al., 2000; Guo et al., 2024; Peng et al., 2024), its relationship with neurodegenerative disorders, particularly PD, remains largely unexplored.

Previous studies on insulin resistance and PD risk have yielded conflicting results, potentially due to variations in study design, insulin sensitivity metrics, and population characteristics. By using eGDR, a validated measure of insulin resistance, this study aims to clarify the metabolic contributions to PD. Understanding the association between eGDR and PD could provide valuable insights into the role of insulin resistance in PD pathogenesis and potentially identify new therapeutic targets. Moreover, given the increasing evidence linking metabolic health to neurodegenerative diseases (Camandola and Mattson, 2017; Dai et al., 2023; Ye et al., 2023; Zhong et al., 2024), investigating whether eGDR could serve as a marker for PD risk or progression has important clinical implications.

Therefore, using data from the National Health and Nutrition Examination Survey (NHANES) 2003–2016, we aimed to: (1) examine the cross-sectional association between eGDR and PD risk; (2) investigate the relationship between eGDR and all-cause mortality in PD patients; and (3) explore potential non-linear relationships in these associations. Given the role of insulin resistance in neurodegeneration, we hypothesize that lower eGDR levels, reflecting greater insulin resistance, will be associated with higher PD risk and mortality.

## 2 Materials and methods

### 2.1 Study design and participants

This analysis utilized data from the National Health and Nutrition Examination Survey (NHANES), a comprehensive national surveillance system implemented biennially since 1999. Conducted by the National Center for Health Statistics (NCHS), NHANES provides nationally representative data on the health and nutritional status of the United States population through standardized interviews and physical examinations. All study protocols received approval from the NCHS Ethics Review Board, and participants provided written informed consent prior to enrollment. The data are publicly accessible through the CDC website^1^.

**FIGURE 1:**
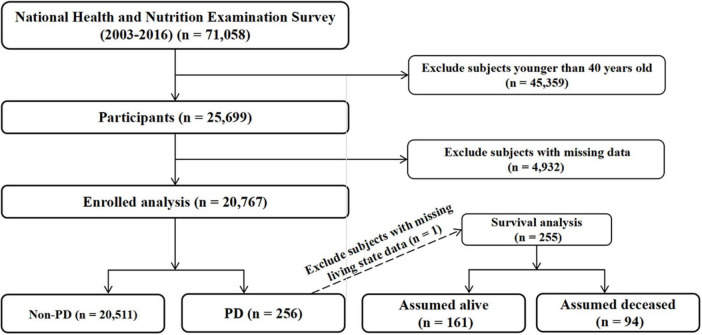
Flow chart of study population selection. Flow chart showing the selection process of study participants from the National Health and Nutrition Examination Survey (NHANES) 2003–2016.

For this study, we included data from seven consecutive NHANES cycles, spanning the years 2003 to 2016. The selection of the study population is depicted in Figure 1. Starting with an initial population of 71,058 participants, we applied the following exclusion criteria: 45,359 subjects younger than 40 years of age and 4,932 subjects with missing data. The final analytic cohort comprised 20,767 participants, including 256 cases with PD and 20,511 non-PD controls. For the survival analysis, one subject was excluded due to missing vital status data, resulting in 255 PD patients (161 alive and 94 deceased at the end of follow-up).

### 2.2 Calculation of eGDR

As previously mentioned, the estimated glucose disposal rate (eGDR) was calculated according to the following equation: eGDR (mg/kg/min) = 21.158 - (0.09 × WC) - (3.407 × HT) - (0.551 × HbA1c) (Epstein et al., 2013). Where WC is waist circumference (cm), HT is hypertension (yes = 1/no = 0), and HbA1c is glycated hemoglobin (%DCCT). Hypertension was defined according to the International Classification of Diseases, 10th Revision (ICD-10) with codes I10, or systolic blood pressure ≥140 mmHg and diastolic blood pressure ≥90 mmHg, or use of antihypertensive medication, or based on general practitioner diagnosis, medication reimbursement, or self-reported information. HbA1c was measured using the High-Performance Liquid Chromatography method. The eGDR values were divided into tertiles: T1 (≥−3.9773 to ≤5.485), T2 (>5.485 to ≤8.408), and T3 (>8.408 to <12.9593). These cut-off values were determined based on the distribution of the data to facilitate the comparison of groups with different eGDR levels in subsequent analyses.

### 2.3 Identification of PD cases

Following established methodologies from previous studies (Liu et al., 2023; Zeng et al., 2023, 2024), we identified PD cases through participants’ medication use data in NHANES. Case classification was based on reported use of anti-parkinsonian medications: individuals reporting the use of these medications were designated as suspected PD cases, while those without documented anti-parkinsonian medication use were classified as non-PD controls.

### 2.4 Assessment of mortality

The primary endpoint of this cohort study was all-cause mortality, encompassing deaths attributed to any cause. Vital status of participants and duration of follow-up were ascertained through linkage to the National Death Index, with surveillance extending through 31 December 2019.1 Follow-up time was calculated from each participant’s initial enrollment in the NHANES program until either their date of death or the end of the study period (31 December 2019), whichever occurred first.

### 2.5 Assessment of other variables

Data on demographic and lifestyle characteristics were collected through standardized questionnaires. Body mass index (BMI) was calculated as weight in kilograms divided by height in meters squared (kg/m^2^). Education level was categorized as below high school, high school, and above high school. Marital status was classified as never married, married/living with partner, and widowed/divorced/separated. Smoking status (yes/no) and alcohol consumption (yes/no) were self-reported. For comorbidities assessment, diabetes was defined by meeting any of the following criteria: self-reported physician diagnosis, glycohemoglobin (HbA1c) ≥6.5%, 2 h oral glucose tolerance test (OGTT) blood glucose ≥11.1 mmol/L, or use of diabetes medication or insulin. Hypertension was diagnosed by self-reported questionnaire data, antihypertensive medication use, or a systolic or diastolic blood pressure reading of ≥140/90 mmHg. Stroke and coronary heart disease were assessed based on self-reported physician diagnosis or relevant medication use.

### 2.6 Statistical analysis

Baseline characteristics were presented as mean (standard error) for continuous variables and frequency (weighted percentage) for categorical variables. Differences between groups were compared using weighted *t*-tests for continuous variables and weighted chi-square tests for categorical variables. The association between eGDR and PD was first examined using weighted univariate logistic regression analysis. Socioeconomic status indicators, such as education level and marital status, may influence individuals’ lifestyles and health conditions, thereby potentially confounding the risk of PD. These variables have been included as confounders for adjustment in previous epidemiological studies on PD (Liu et al., 2023; Zeng et al., 2023, 2024). Weighted multivariate logistic regression analyses were then conducted with four progressive adjustment models: Crude model: no adjustment for any confounding factors; Model 1: adjusted for age, gender, race, BMI, and height; Model 2: adjusted for Model 1 variables plus education, marital status, alcohol use, and smoking status; Model 3: adjusted for Model 2 variables plus stroke, coronary heart disease, and diabetes mellitus. Stratified analyses were performed to assess potential effect modifications by age, gender, race, and comorbidities. For survival analysis, weighted Cox proportional hazards models were employed using the same adjustment strategy. Kaplan-Meier curves were generated to visualize survival probabilities across eGDR tertiles, with differences assessed using the log-rank test. Restricted cubic spline analyses were conducted to examine potential non-linear relationships between eGDR and both PD risk and mortality. All statistical analyses were performed using R software version 4.2.2. A two-sided *P*-value < 0.05 was considered statistically significant.

## 3 Results

### 3.1 Baseline characteristics of study participants according to Parkinson’s disease status

As shown in Table 1, a total of 20,741 participants were included in this analysis, among whom 255 participants had PD. PD patients were significantly older than non-PD participants (60.644 ± 1.178 vs 57.304 ± 0.162 years, *P* = 0.005). There was a significant gender difference in PD distribution, with females accounting for a higher proportion of PD cases (62.481% vs 52.201%, *P* = 0.011). Race distribution also varied significantly between groups (*P* = 0.004), with Non-Hispanic Whites comprising the majority of PD cases (83.101%). The mean eGDR was significantly lower in PD patients compared to non-PD participants (6.476 ± 0.205 vs 7.174 ± 0.035, *P* = 0.001). When categorized into tertiles, there was a significant difference in eGDR distribution (*P* < 0.001), with a higher proportion of PD cases in the lower tertiles (T1: 38.264%, T2: 37.892%) compared to the highest tertile (T3: 23.844%). Regarding comorbidities, PD patients had a significantly higher prevalence of stroke (12.356% vs 3.924%, *P* < 0.0001) and diabetes (24.634% vs 18.040%, *P* = 0.016). Other characteristics, including BMI, height, marital status, education level, alcohol use, smoking status, and coronary heart disease, showed no significant differences between groups.

**TABLE 1:** Baseline characteristics of study participants according to Parkinson’s disease status.

Variable	Total	Non-PD	PD	*P*-value
Age	57.345 (0.163)	57.304 (0.162)	60.644 (1.178)	**0.005**
Gender				**0.011**
Male	10,237 (47.675)	10,119 (47.799)	118 (37.519)	–
Female	10,504 (52.325)	10,367 (52.201)	137 (62.481)	–
Race				**0.004**
Non-Hispanic Black	4,186 (9.683)	4,149 (9.708)	37 (7.621)	–
Non-Hispanic White	9,975 (74.739)	9,811 (74.637)	164 (83.101)	–
Mexican American	3,222 (5.983)	3,196 (6.008)	26 (3.945)	–
Other race	3,358 (9.596)	3,330 (9.648)	28 (5.334)	–
BMI	29.222 (0.076)	29.211 (0.077)	30.135 (0.532)	0.093
Height	168.234 (0.117)	168.252 (0.119)	166.757 (0.852)	0.087
Marital				0.457
Never married	1,599 (6.784)	1568 (6.764)	31 (8.394)	–
Married/living with partner	13,035 (68.463)	12,890 (68.511)	145 (64.500)	–
Widowed/divorced/separated	6,107 (24.753)	6,028 (24.725)	79 (27.105)	–
Education				0.572
High education	7,814 (34.481)	7,710 (34.447)	104 (37.258)	–
Below high education	2,848 (6.485)	2,815 (6.476)	33 (7.177)	–
Over high education	10,079 (59.035)	9,961 (59.077)	118 (55.565)	–
Alcohol user				0.468
No	3,128 (11.411)	3,096 (11.431)	32 (9.773)	–
Yes	17,613 (88.589)	17,390 (88.569)	223 (90.227)	–
Smoke				0.693
No	10,418 (50.371)	10,293 (50.353)	125 (51.859)	–
Yes	10,323 (49.629)	10,193 (49.647)	130 (48.141)	–
eGDR	7.165 (0.035)	7.174 (0.035)	6.476 (0.205)	**0.001**
eGDR				**< 0.001**
T1	6,912 (29.641)	6,805 (29.536)	107 (38.264)	–
T2	6,924 (31.801)	6,828 (31.727)	96 (37.892)	–
T3	6,905 (38.558)	6,853 (38.737)	52 (23.844)	–
Stroke				**< 0.0001**
No	19,649 (95.975)	19,428 (96.076)	221 (87.644)	–
Yes	1,092 (4.025)	1,058 (3.924)	34 (12.356)	–
Coronary heart disease				0.371
No	19,476 (94.780)	19,244 (94.799)	232 (93.211)	–
Yes	1,265 (5.220)	1,242 (5.201)	23 (6.789)	–
Diabetes				**0.016**
No	15,782 (81.881)	15,608 (81.960)	174 (75.366)	–
Yes	4,959 (18.119)	4,878 (18.040)	81 (24.634)	–

Data are presented as mean (standard error) for continuous variables and n (weighted%) for categorical variables. *P*-values were calculated using weighted *t*-tests for continuous variables and weighted chi-square tests for categorical variables. BMI, body mass index; eGDR, estimated glucose disposal rate; T1, T2, T3: tertiles of eGDR. Statistically significant results are highlighted in bold.

### 3.2 Univariate logistic regression analysis of factors associated with Parkinson’s disease

In the univariate logistic regression analysis, several factors were significantly associated with PD. As shown in Table 2, age showed a positive association with PD (OR: 1.023, 95% CI: 1.008–1.039, *P* = 0.003). In lay terms, this means that for every additional year of age, the odds of having PD increase by approximately 2.3%. Female gender was associated with higher odds of PD compared to males (OR: 1.525, 95% CI: 1.102–2.110, *P* = 0.011). The analysis of eGDR revealed a significant inverse association with PD, both as a continuous variable (OR: 0.907, 95% CI: 0.856–0.960, *P* = 0.001) and when categorized into tertiles. Compared to the lowest tertile (T1), the highest tertile (T3) showed significantly lower odds of PD (OR: 0.475, 95% CI: 0.311–0.726, *P* < 0.001), meaning that individuals in the highest eGDR tertile had about 52.5% lower odds of having PD compared to those in the lowest tertile, while the middle tertile (T2) showed no significant difference (OR: 0.922, 95% CI: 0.643–1.321, *P* = 0.655). Regarding comorbidities, stroke showed the strongest association with PD, with stroke patients having more than three times the odds of having PD (OR: 3.452, 95% CI: 2.150–5.541, *P* < 0.0001). Diabetes was also significantly associated with increased odds of PD (OR: 1.485, 95% CI: 1.077–2.048, *P* = 0.016). Other variables, including race, BMI, height, marital status, education level, alcohol use, smoking status, and coronary heart disease, did not show statistically significant associations with PD in the univariate analysis.

**TABLE 2:** Univariate logistic regression analysis of factors associated with Parkinson’s disease.

Variable	OR (95% CI)	*P*-value
Age	1.023 (1.008,1.039)	**0.003**
**Gender**		
Male	Ref	Ref
Female	1.525 (1.102,2.110)	**0.011**
**Race**		
Non-Hispanic Black	Ref	Ref
Non-Hispanic White	1.418 (0.995,2.021)	0.053
Mexican American	0.836 (0.513,1.364)	0.471
Other race	0.704 (0.380,1.306)	0.263
BMI	1.021 (0.998,1.044)	0.071
Height	0.985 (0.969,1.002)	0.090
**Marital**		
Never married	Ref	Ref
Married/living with partner	0.759 (0.442,1.302)	0.313
Widowed/ divorced/ separated	0.883 (0.521,1.498)	0.643
**Education**		
High education	Ref	Ref
Below high education	1.025 (0.632,1.661)	0.921
Over high education	0.870 (0.607,1.246)	0.443
**Alcohol user**		
No	Ref	Ref
Yes	1.192 (0.739,1.921)	0.469
**Smoke**		
No	Ref	Ref
Yes	0.941 (0.696,1.274)	0.693
eGDR	0.907 (0.856,0.960)	0.001
**eGDR**		
T1	Ref	Ref
T2	0.922 (0.643,1.321)	0.655
T3	0.475 (0.311,0.726)	**< 0.001**
**Stroke**		
No	Ref	Ref
Yes	3.452 (2.150,5.541)	**<0.0001**
**Coronary heart disease**		
No	Ref	Ref
Yes	1.328 (0.709,2.487)	0.373
**Diabetes**		
No	Ref	Ref
Yes	1.485 (1.077,2.048)	**0.016**

Data are presented as odds ratios (OR) with 95% confidence intervals (CI). All analyses were conducted using weighted logistic regression. ref, reference category; BMI, body mass index; eGDR, estimated glucose disposal rate; T1, T2, T3: tertiles of eGDR. Statistically significant results are highlighted in bold.

### 3.3 Association between eGDR and Parkinson’s disease in different adjustment models

The association between eGDR and PD was examined using four progressive adjustment models in Table 3. In the crude model, eGDR showed a significant inverse association with PD risk (OR: 0.906, 95% CI: 0.856–0.960, *P* < 0.001). This association remained significant after adjusting for demographic and anthropometric factors in Model 1 (OR: 0.906, 95% CI: 0.830–0.988, *P* = 0.027) and further adjustment for lifestyle factors in Model 2 (OR: 0.905, 95% CI: 0.829–0.989, *P* = 0.028). However, after additional adjustment for comorbidities in Model 3, the association was attenuated and became non-significant (OR: 0.926, 95% CI: 0.840–1.020, P = 0.117). When analyzing eGDR in tertiles, compared to the lowest tertile (T1), the highest tertile (T3) consistently showed lower odds of PD across all models. The association remained significant even in the fully adjusted Model 3 (OR: 0.574, 95% CI: 0.337–0.976, P = 0.040), indicating that individuals in the highest tertile had about 42.6% lower odds of having PD compared to those in the lowest tertile. The middle tertile (T2) showed no significant difference from T1 in any model. The trend analysis across tertiles demonstrated a significant linear trend in all models (P for trend = 0.033 in Model 3), suggesting a dose-response relationship between eGDR levels and PD risk.

**TABLE 3:** Association between eGDR and Parkinson’s disease in different adjustment models.

Character	Crude model		Model 1		Model 2		Model 3	
	**OR (95% CI)**	***P*-value**	**OR (95% CI)**	***P*-value**	**OR (95% CI)**	***P*-value**	**OR (95% CI)**	***P*-value**
eGDR	0.906 (0.856, 0.960)	**< 0.001**	0.906 (0.830, 0.988)	**0.027**	0.905 (0.829, 0.989)	**0.028**	0.926 (0.840, 1.020)	0.117
T1	Ref	–	Ref	–	Ref	–	Ref	–
T2	0.924 (0.646, 1.323)	0.665	0.921 (0.635, 1.336)	0.661	0.918 (0.630, 1.337)	0.652	0.975 (0.667, 1.425)	0.893
T3	0.474 (0.310, 0.725)	**< 0.001**	0.520 (0.316, 0.857)	**0.011**	0.518 (0.313, 0.857)	**0.011**	0.574 (0.337, 0.976)	**0.040**
P for trend	–	**< 0.001**	–	**0.008**	–	**0.008**	–	**0.033**

Data are presented as odds ratios (OR) with 95% confidence intervals (CI). Model 1 adjusted for age, gender, race, BMI, and height. Model 2 adjusted for variables in Model 1 plus education, marital status, alcohol use, and smoking status. Model 3 adjusted for variables in Model 2 plus stroke, coronary heart disease, and diabetes mellitus. eGDR, estimated Glucose Disposal Rate; T1, T2, T3, tertiles of eGDR (T1, lowest tertile as reference). Statistically significant results are highlighted in bold.

### 3.4 Stratified analysis of the association between eGDR and Parkinson’s disease

Stratified analyses were performed to examine whether the association between eGDR and PD varied across different subgroups. The analyses revealed several notable patterns while adjusting for marital status, education, height, and BMI. As shown in Table 4, age-stratified analysis showed a stronger association in the 40–60 years age group (OR: 0.860, 95% CI: 0.756–0.978, *P* = 0.022) compared to those over 60 years (OR: 0.940, 95% CI: 0.837–1.056, *P* = 0.295), although the interaction was not significant (P for interaction = 0.139). The association was significant in both males (OR: 0.840, 95% CI: 0.722–0.978, *P* = 0.025) and females (OR: 0.889, 95% CI: 0.800–0.989, *P* = 0.031), with no significant gender interaction (P for interaction = 0.312). In race-stratified analyses, significant associations were observed among Non-Hispanic Whites (OR: 0.884, 95% CI: 0.795–0.982, *P* = 0.023) and Mexican Americans (OR: 0.790, 95% CI: 0.652–0.958, *P* = 0.017), but not among Non-Hispanic Blacks (OR: 0.962, 95% CI: 0.779–1.187, *P* = 0.715) or Other Races (OR: 0.905, 95% CI: 0.755–1.085, *P* = 0.279). However, the interaction by race was not significant (P for interaction = 0.339). When stratified by comorbidities, the association was significant among non-diabetic participants (OR: 0.879, 95% CI: 0.783–0.986, *P* = 0.029) but not among diabetic participants (OR: 0.928, 95% CI: 0.816–1.055, *P* = 0.251). Similar patterns were observed for other comorbidities, with stronger associations generally observed in participants without the respective conditions, although none of the interaction tests reached statistical significance.

**TABLE 4:** Stratified analysis of the association between eGDR and Parkinson’s disease.

Character	OR (95% CI)	*P*-value	P for interaction
Age			0.139
40–60 years old	0.860 (0.756,0.978)	**0.022**	–
>60 years old	0.940 (0.837,1.056)	0.295	–
Gender			0.312
Male	0.840 (0.722,0.978)	**0.025**	–
Female	0.889 (0.800,0.989)	**0.031**	–
Race			0.339
Non-Hispanic Black	0.962 (0.779,1.187)	0.715	–
Non-Hispanic White	0.884 (0.795,0.982)	**0.023**	–
Mexican American	0.790 (0.652,0.958)	**0.017**	–
Other race	0.905 (0.755,1.085)	0.279	–
Alcohol user			0.874
No	0.926 (0.731,1.172)	0.519	–
Yes	0.877 (0.800,0.962)	**0.006**	–
Smoke			0.904
No	0.885 (0.794,0.987)	**0.029**	–
Yes	0.877 (0.772,0.997)	**0.045**	–
Stroke			0.995
No	0.915 (0.834,1.004)	0.062	–
Yes	0.800 (0.637,1.005)	0.055	–
Coronary heart disease			0.449
No	0.888 (0.811,0.973)	**0.011**	–
Yes	0.870 (0.684, 1.106)	0.252	–
Hypertension			0.211
No	0.861 (0.672,1.102)	0.232	–
Yes	0.849 (0.705,1.023)	0.085	–
Diabetes			0.218
No	0.879 (0.783,0.986)	**0.029**	–
Yes	0.928 (0.816,1.055)	0.251	–

Data are presented as odds ratios (OR) with 95% confidence intervals (CI) for eGDR (per unit increase). All models were adjusted for marital status, education, height, and BMI. P for interaction was calculated using multiplicative interaction terms in the adjusted models. eGDR, estimated glucose disposal rate. Statistically significant results are highlighted in bold.

### 3.5 Association between eGDR and all-cause mortality among Parkinson’s disease patients in cox regression models

The association between eGDR and all-cause mortality among PD patients was evaluated using four progressive Cox regression models. As shown in Table 5, in the crude model, higher eGDR was significantly associated with lower mortality risk (HR: 0.876, 95% CI: 0.806–0.953, *P* = 0.002). This means that for every unit increase in eGDR, the risk of mortality decreased by about 12.4%. This protective association remained robust and even strengthened after adjusting for demographic and anthropometric factors in Model 1 (HR: 0.835, 95% CI: 0.746–0.934, *P* = 0.002) and after further adjustment for lifestyle factors in Model 2 (HR: 0.845, 95% CI: 0.756–0.944, *P* = 0.003). The association persisted, though slightly attenuated, in the fully adjusted Model 3, which included additional adjustment for comorbidities (HR: 0.875, 95% CI: 0.775–0.987, *P* = 0.030). When analyzing eGDR in tertiles, compared to the lowest tertile (T1), participants in the highest tertile (T3) consistently showed significantly lower mortality risk across all models. The association remained strong in the fully adjusted Model 3 (HR: 0.330, 95% CI: 0.141–0.768, *P* = 0.010), suggesting that individuals in the highest tertile had a 67% lower risk of all-cause mortality compared to those in the lowest tertile. The middle tertile (T2) showed no significant difference from the reference group across all models (Model 3 HR: 1.185, 95% CI: 0.635–2.210, *P* = 0.594), indicating a potential threshold effect in the association between eGDR and mortality risk.

**TABLE 5:** Association between eGDR and all-cause mortality among Parkinson’s disease patients in cox regression models.

Character	Crude model		Model 1		Model 2		Model 3	
	**HR (95% CI)**	***P*-value**	**HR (95% CI)**	***P*-value**	**HR (95% CI)**	***P*-value**	**HR (95% CI)**	***P*-value**
eGDR	0.876 (0.806, 0.953)	**0.002**	0.835 (0.746, 0.934)	**0.002**	0.845 (0.756, 0.944)	**0.003**	0.875 (0.775, 0.987)	**0.030**
T1	Ref	Ref	Ref	Ref	Ref	Ref	Ref	Ref
T2	1.045 (0.600, 1.819)	0.877	0.961 (0.566, 1.631)	0.882	1.012 (0.551, 1.858)	0.969	1.185 (0.635, 2.210)	0.594
T3	0.284 (0.122, 0.663)	**0.004**	0.316 (0.135, 0.741)	**0.008**	0.324 (0.141, 0.744)	**0.008**	0.330 (0.141, 0.768)	**0.010**

Data are presented as hazard ratios (HR) with 95% confidence intervals (CI). Model 1 adjusted for age, gender, race, BMI, and height. Model 2 adjusted for variables in Model 1 plus education, marital status, alcohol use, and smoking status. Model 3 adjusted for variables in Model 2 plus stroke, coronary heart disease, and diabetes mellitus. eGDR, estimated glucose disposal rate; T1, T2, T3, tertiles of eGDR (T1, lowest tertile as reference). Statistically significant results are highlighted in bold.

### 3.6 Kaplan-Meier survival curves for all-cause mortality stratified by eGDR tertiles

**FIGURE 2:**
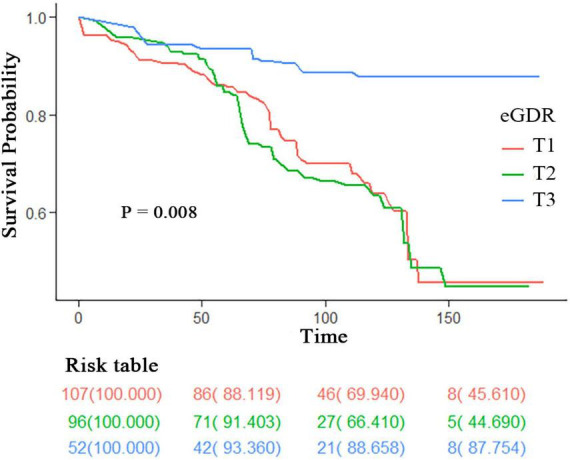
Kaplan-Meier survival curves for all-cause mortality stratified by eGDR tertiles. Survival curves are stratified by eGDR tertiles (T1: lowest tertile; T2: middle tertile; T3: highest tertile). The risk table shows the number of participants at risk (survival probability %) at 0, 50, 100, and 150 months. *P*-value was calculated using the log-rank test. eGDR, estimated glucose disposal rate.

Figure 2 demonstrates the Kaplan-Meier survival curves for all-cause mortality among PD patients stratified by eGDR tertiles, with a significant difference observed between groups (log-rank *P* = 0.008). The highest tertile (T3, blue line) showed consistently better survival throughout the follow-up period compared to the middle (T2, green line) and lowest tertiles (T1, red line). The survival probability at different time points can be tracked through the risk table: At baseline (0 months): T1 started with 107 patients, T2 with 96 patients, and T3 with 52 patients (all with 100% survival); At 50 months: T1 retained 86 patients (88.119% survival), T2 retained 71 patients (91.403% survival), and T3 retained 42 patients (93.360% survival); At 100 months: T1 had 46 patients (69.940% survival), T2 had 27 patients (68.410% survival), and T3 had 21 patients (88.658% survival); At 150 months: T1 had 8 patients (45.610% survival), T2 had five patients (44.690% survival), and T3 had eight patients (87.754% survival). The survival curves demonstrated a clear separation, with T3 maintaining the highest survival probability throughout the follow-up period, while T1 and T2 showed similar but lower survival probabilities, particularly after 100 months of follow-up.

### 3.7 Non-linear association of eGDR with Parkinson’s disease risk and all-cause mortality

**FIGURE 3:**
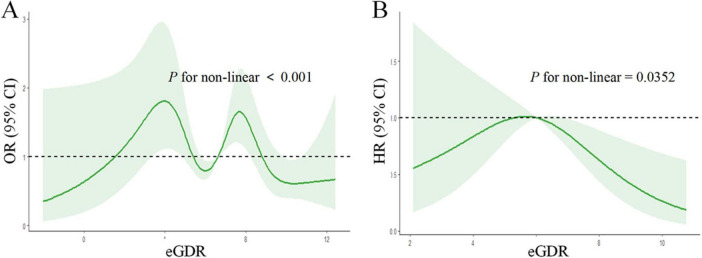
Non-linear association of eGDR with Parkinson’s disease risk and all-cause mortality. The relationship between eGDR and outcomes was assessed using restricted cubic spline analyses with adjustment for potential confounders. The solid green lines represent the estimated odds ratios (OR) or hazard ratios (HR), and the shaded areas represent the 95% confidence intervals. The reference line (OR/HR = 1.0) is indicated by the dashed horizontal line. **(A)** Non-linear Association of eGDR with PD Risk (OR). **(B)** Non-linear Association of eGDR with All-cause Mortality (HR). *P*-value < 0.05 for non-linearity indicates a statistically significant non-linear relationship. eGDR, estimated glucose disposal rate.

Figure 3 illustrates the complex non-linear relationships between eGDR and PD outcomes through restricted cubic spline analyses. Panel A demonstrates a significant M-shaped non-linear association between eGDR and PD risk (P for non-linearity < 0.001). The relationship curve shows two distinct peaks: one occurring around an eGDR value of four, and another at approximately 7–8. After these peaks, the risk of PD gradually decreases with increasing eGDR values. The 95% confidence intervals (shown in green shading) become wider at the extremes of eGDR values, indicating increased uncertainty in these regions. Panel B reveals an inverted U-shaped relationship between eGDR and all-cause mortality risk among PD patients (P for non-linearity = 0.0352). The HR curve shows an initial increase in mortality risk up to an eGDR value of approximately 5–6, followed by a steady decline in risk at higher eGDR values. The mortality risk appears lowest at the highest eGDR values (> 8), suggesting a potentially protective effect of higher eGDR levels against mortality in PD patients. The confidence intervals also widen at the extremes, particularly at lower eGDR values.

## 4 Discussion

### 4.1 Epidemiological implications

Our study demonstrated a significant association between eGDR and both PD risk and mortality outcomes using NHANES 2003–2016 data. Several important findings emerged from this analysis. First, we found that higher eGDR levels were associated with lower odds of PD, with the highest tertile showing significantly reduced risk compared to the lowest tertile. Second, we observed a complex M-shaped non-linear relationship between eGDR and PD risk. Third, among PD patients, higher eGDR levels were associated with better survival outcomes, displaying an inverted U-shaped relationship with mortality risk.

### 4.2 Potential mechanistic insights

Parkinson’s disease is currently considered a disease influenced by both environmental and genetic factors. In fact, pathological degeneration in PD patients’ brains begins 10–20 years before clinical diagnosis (Hilker et al., 2005). By the time patients show obvious motor symptoms, about 50% of dopaminergic neurons in the substantia nigra are already lost (Reeve et al., 2014). Some early potential pathophysiological mechanisms play important roles in the early clinical stages, and overlapping disease mechanisms can be found between diabetes and PD. Diabetes is a metabolic disease primarily characterized by elevated peripheral blood glucose (American Diabetes Association, 2011). Similar to PD, T2DM is an age-related disease showing a trend toward younger onset (Kwak et al., 2010). Epidemiological studies both domestically and internationally have noted potential connections between diabetes and PD, with diabetes being a potential risk factor for PD (Cheong et al., 2020).

Sandyk first noticed the relationship between diabetes and PD in 1993, observing that PD patients with diabetes more commonly experienced worsening motor symptoms and responded poorly to treatment (Xu et al., 2011). Subsequently, numerous epidemiological studies confirmed a positive correlation between PD and diabetes. For example, a Finnish study of 51,552 people showed that T2DM patients had an 85% higher risk of developing PD compared to the general population. While other studies reported different figures, they confirmed that diabetes could increase PD risk by 28%–40% (Xu et al., 2011). In the past decade, two meta-analyses provided evidence in this field. The first meta-analysis in 2011 identified diabetes as a risk factor for PD (Cereda et al., 2011). The second study in 2016, which included seven cohort studies involving over 1.7 million people, concluded that diabetes patients had approximately 38% increased risk of PD (Yue et al., 2016). Recent Mendelian randomization studies have also revealed a causal relationship between diabetes and PD at the genetic level, confirming diabetes as a risk factor for PD development (Chohan et al., 2021). Additionally, studies have shown that diabetes itself can worsen both motor and non-motor symptoms of PD (Cereda et al., 2012; Kotagal et al., 2013), such as gait and posture abnormalities or severe cognitive impairment (Bohnen et al., 2014; Bosco et al., 2012; Giuntini et al., 2014), with diabetes advancing the onset of PD motor complications by nearly 1 year (Mohamed Ibrahim et al., 2018). Recent research has also found that pre-diabetes or mild blood glucose elevation in non-diabetic populations may increase PD risk (Rhee et al., 2020; Sánchez-Gómez et al., 2021). Our research similarly observed a higher proportion of diabetes in the PD group compared to the non-PD group, with univariate logistic regression analysis indicating diabetes as one of the potential risk factors for PD. These epidemiological studies reveal a close relationship between diabetes and PD, suggesting that diabetes may play a role in early PD or that there are commonalities in their pathophysiological mechanisms.

IR is the core characteristic of T2DM, a concept first proposed by Himsworth (2011) in 1936, defined as “decreased sensitivity and responsiveness of insulin target organs or tissues to insulin, or impaired insulin signal transduction.” The occurrence of IR is related to both genetic and environmental factors, with obesity being the main environmental risk factor. As the global obese population continues to expand, the prevalence of IR is steadily rising (Kahn et al., 2006). From a microscopic perspective, IR primarily results from abnormal insulin signaling pathway transduction. The serine residues at positions 616 and 312 of insulin receptor substrate IRS-1 protein are two key phosphorylation sites. Phosphorylation at position 616 inhibits the AKT signaling pathway, reducing insulin signal transduction, while phosphorylation at position 312 causes IRS-1 to escape cytoplasmic degradation, reducing IRS-1 expression. Changes in these key proteins or phosphorylation levels related to insulin signal transduction pathways can lead to IR (Demir et al., 2021). Some studies have found connections between IR-related indicator abnormalities and PD, such as elevated blood glucose levels (Rhee et al., 2020; Sánchez-Gómez et al., 2021). Earlier research showed that 50%–80% of PD patients have abnormal glucose tolerance (Sandyk, 1993). Additionally, some epidemiological studies indicate that high BMI is associated with increased PD risk (Abbott et al., 2002; Jeong et al., 2020), and increased waist circumference also raises PD risk (Park et al., 2022; Riso et al., 2019). IR is also associated with more severe PD phenotypes, accelerated disease progression, and increased risk of PD dementia (Cereda et al., 2012; Kotagal et al., 2013). At the molecular level, brain IR mainly manifests as impaired downstream signal transduction after insulin receptor activation (Arnold et al., 2018), with evidence of insulin receptor loss in the substantia nigra of PD patients found early on Moroo et al. (1994), Takahashi et al. (1996). In PD patients’ substantia nigra pathological samples, significant loss of IRS-1 mRNA and increased IR were detected (Takahashi et al., 1996). Elevated phosphorylation levels of serine residues (which inactivate insulin signal transduction) (IRS-312) were found in the basal ganglia and substantia nigra, with these changes occurring before dopaminergic neuron damage (Moroo et al., 1994). In PD patients and animal models’ brain tissue, the ratio of phosphorylated AKT to total AKT was significantly reduced (Malagelada et al., 2008; Selvakumar et al., 2020). GSK-3β is one of AKT’s activation targets; reduced GSK3β after AKT activation increases cellular autophagy to clear abnormally aggregated α-synuclein, potentially reducing dopaminergic neuron apoptosis, however, increased GSK3β expression was found in PD patients and animal models (Hernandez-Baltazar et al., 2013). Additionally, basic research has demonstrated that mice with brain IR show impaired dopamine transport function (Sharma and Taliyan, 2018), and MPTP-modeled mice show impaired insulin signaling in the substantia nigra (Bousquet et al., 2012; Kao et al., 2019), with diabetes and PD sharing common pathway damage (Labandeira et al., 2022). Furthermore, mice fed high-fat diets to induce insulin signaling impairment and diabetic mice showed increased sensitivity to 6-OHDA and MPTP, leading to significantly increased nigrostriatal degeneration and reduced dopaminergic signaling (Labandeira et al., 2022; Morris et al., 2010; Morris et al., 2011a; Morris et al., 2011b; Wang et al., 2014). In fact, the insulin signaling pathway balances normal dopaminergic neuron function, with mTOR being the main downstream substrate of the IRS-1/AKT pathway. In PD patients and animal models, blockage of this pathway leads to reduced autophagy function and α-syn aggregation (Heras-Sandoval et al., 2014). Using the mTORC1 inhibitor rapamycin can reduce α-syn aggregation in PD animal models (Stoica et al., 2011). Experimental model results indicate that inhibiting AKT signal transduction leads to dopaminergic cell death (Xu et al., 2014). We found that higher eGDR levels are associated with lower PD probability, with the highest quartile showing significantly reduced risk compared to the lowest quartile. This finding suggests that better insulin sensitivity may have a protective effect against PD. This association is consistent with previous studies indicating that insulin resistance and related metabolic dysfunction may lead to neurodegeneration.

Considering the above evidence, these signs reveal that IR participates in PD’s pathogenesis process, or IR serves as a susceptible background for PD, with IR’s role in neurodegeneration acting not only as a contributing factor to disease onset but also as a modifier of motor and non-motor symptoms. We observed a complex M-shaped non-linear relationship between eGDR and PD risk. This new finding suggests that the relationship between insulin sensitivity and Parkinson’s disease is more complex than previously thought. The observed pattern may reflect different pathophysiological mechanisms operating at different levels of insulin sensitivity. In PD patients, higher eGDR levels are associated with better survival outcomes, showing an inverted U-shaped relationship with mortality risk. This finding extends previous research on diabetes and PD progression. The observed relationship suggests an optimal range of insulin sensitivity for PD patient survival, with both very low and very high levels potentially associated with poorer outcomes. This pattern may reflect complex interactions between metabolism, aging, and neurodegeneration.

In our study, we adjusted for several potential confounding variables, including age, gender, BMI, and comorbidities such as diabetes, stroke, and coronary heart disease, to better isolate the association between eGDR and PD risk. Age is a known risk factor for PD, and obesity, measured by BMI, is linked to insulin resistance and PD risk. By adjusting for these factors, we were able to demonstrate that the association between eGDR and PD risk remained significant, suggesting that eGDR may be an independent risk factor for PD. However, we did not directly measure genetic predisposition in this study, and we acknowledge that it may play a role in the development of PD and its association with metabolic factors. Future studies should aim to include genetic data to better understand the complex interplay between genetics, metabolism, and PD risk.

### 4.3 Strengths and limitations

The strengths of our study include its large, nationally representative sample, comprehensive adjustment for potential confounders, and the novel investigation of both cross-sectional and longitudinal relationships between eGDR and PD. The use of restricted cubic spline analysis allowed us to detect important non-linear relationships that might have been missed using traditional analytical approaches. However, several limitations should be considered when interpreting our results. First, the cross-sectional nature of the primary analysis limits our ability to establish causality between eGDR and PD risk. Second, PD cases were identified through medication use, which might have led to misclassification of some cases. Third, in our study, we identified age, gender, diabetes, and stroke as significant factors associated with PD risk in univariate analyses. Despite adjusting for these factors in our multivariate models, eGDR remained significantly associated with PD, highlighting its potential as an independent risk factor. Additionally, stratified analyses revealed no significant interactions between these factors and eGDR, further supporting its independent association. However, we acknowledge that residual confounding or the influence of unmeasured variables cannot be entirely ruled out. For instance, lifestyle factors such as diet, physical activity, and socioeconomic status, which were not fully captured in our analysis, could independently influence both insulin resistance and PD risk. Additionally, genetic factors and inflammatory markers, which were not assessed in this study, may also play a role in the observed association. Future research should aim to incorporate these factors to provide a more nuanced understanding of the relationship between eGDR and PD. While our findings suggest that eGDR is an independent risk factor for PD, further investigation is needed to elucidate the potential influence of residual confounders and unmeasured variables. Fourth, the formula for eGDR was originally developed in type 1 diabetes patients, and its validity in the general population requires further validation.

### 4.4 Clinical implications

In clinical practice, the significant associations between eGDR and both PD risk and all-cause mortality highlight its potential utility as a biomarker and risk stratification tool. For example, eGDR could be incorporated into existing PD screening protocols to identify individuals at higher risk of developing PD. Clinicians could use eGDR to prioritize patients for more frequent monitoring or early interventions, such as lifestyle modifications (e.g., dietary changes, increased physical activity) or targeted therapies aimed at improving insulin sensitivity. Additionally, eGDR could be used to tailor prevention strategies or treatment plans based on an individual’s glucose metabolism status. For instance, patients with low eGDR values might benefit from interventions to improve insulin resistance, which could potentially reduce their risk of PD progression or all-cause mortality. Furthermore, eGDR could be included in risk assessment models to provide a more comprehensive evaluation of PD risk and inform clinical decision-making. Future studies should explore the feasibility and effectiveness of integrating eGDR into clinical practice to validate its role in PD risk prediction and management. Additionally, research is needed to understand the biological mechanisms underlying the observed associations, particularly the non-linear relationships identified in our analysis. While higher eGDR levels were associated with better survival outcomes in PD patients, it is unclear whether this reflects slower disease progression or simply lower mortality. Future longitudinal studies with detailed clinical assessments of PD progression are needed to determine whether eGDR affects the rate of PD progression. If eGDR is found to be associated with slower disease progression, this would make the findings more clinically meaningful and suggest that interventions targeting insulin sensitivity could potentially slow the progression of PD. In the meantime, the observed association between higher eGDR levels and lower mortality risk suggests that eGDR may be a useful marker for predicting outcomes in PD patients. Further research is needed to explore the underlying mechanisms and potential clinical applications of these findings.

## 5 Conclusion

In this large, nationally representative study, we found that higher eGDR levels were associated with lower PD risk and better survival among PD patients, suggesting a potential role for insulin sensitivity in PD pathogenesis and progression. The complex non-linear relationships observed between eGDR and both PD risk and mortality highlight the intricate nature of metabolic factors in neurodegenerative diseases. These findings suggest that maintaining metabolic health might be important in PD prevention and management, though further research is needed to confirm these associations and explore potential therapeutic implications.

## Data Availability

Publicly available datasets were analyzed in this study. This data can be found here: https://www.cdc.gov/nchs/nhanes/index.htm.

## Footnotes

1
https://www.cdc.gov/nchs/nhanes/index.htm
